# The splicing factor SR2 is an important virulence factor of *Toxoplasma gondii*

**DOI:** 10.3389/fmicb.2023.1302512

**Published:** 2023-11-23

**Authors:** Xiao-Jing Wu, Jin Gao, Xiao-Nan Zheng, Hany M. Elsheikha, Ting-Ting Li, Yong-Jie Kou, Meng Wang, Xing-Quan Zhu

**Affiliations:** ^1^Laboratory of Parasitic Diseases, College of Veterinary Medicine, Shanxi Agricultural University, Taigu, Shanxi Province, China; ^2^State Key Laboratory for Animal Disease Control and Prevention, Key Laboratory of Veterinary Parasitology of Gansu Province, Lanzhou Veterinary Research Institute, Chinese Academy of Agricultural Sciences, Lanzhou, Gansu Province, China; ^3^Faculty of Medicine and Health Sciences, School of Veterinary Medicine and Science, University of Nottingham, Sutton Bonington Campus, Loughborough, United Kingdom

**Keywords:** *Toxoplasma gondii*, SR proteins, splicing factor SR2, alternative splicing, pathogenicity, CRISPR-Cas9

## Abstract

Serine/arginine-rich (SR) proteins are key factors with important roles in constitutive and alternative splicing (AS) of pre-mRNAs. However, the role of SR splicing factors in the pathogenicity of *T. gondii* remains largely unexplored. Here, we investigated the role of splicing factor SR2, a homolog of *Plasmodium falciparum* SR1, in the pathogenicity of *T. gondii*. We functionally characterized the predicted SR2 in *T. gondii* by gene knockout and studied its subcellular localization by endogenous protein HA tagging using CRISPR-Cas9 gene editing. The results showed that SR2 was localized in the nucleus and expressed in the tachyzoite and bradyzoite stages. *In vitro* studies including plaque formation, invasion, intracellular replication, egress and bradyzoite differentiation assays showed that deletion of SR2 in type I RH strain and type II Pru strains had no significant effect on the parasite growth and bradyzoite differentiation (*p* > 0.05). Interestingly, the disruption of SR2 in RH type I (*p* < 0.0001) and Pru type II (*p* < 0.05) strains resulted in varying degrees of attenuated virulence. In addition, disruption of SR2 in type II Pru strain significantly reduced brain cyst burden by ~80% (*p* < 0.0001). Collectively, these results suggest that splicing factor SR2 is important for the pathogenicity of *T. gondii*, providing a new target for the control and treatment of toxoplasmosis.

## 1 Introduction

*Toxoplasma gondii* is an obligate intracellular apicomplexan parasite which infects many warm-blooded animals and humans. It is estimated to chronically infect one third of the world’s population, causing public health and socio-economic impacts (Montoya and Liesenfeld, 2004; Milne et al., 2020). Although infection is asymptomatic in immunocompetent individuals, the reactivation of a dormant *T. gondii* infection in immunocompromised individuals such as HIV and organ transplant recipients may lead to severe and life-threatening consequences (Elsheikha et al., 2021). In addition, congenital infection can lead to miscarriages or mental and visual impairment (Smith et al., 2021). There is a clinical need to explore new targets for therapeutic development.

*T. gondii* has a remarkable ability to respond to and thrive in various environments. The success of *T. gondii* is partly facilitated by its ability to reprogram its gene expression at the transcriptional and post-transcriptional levels. Alternative splicing (AS) is a crucial cellular process which allows a single gene to produce multiple mRNA transcripts and, consequently, expand the protein repertoire encoded by the genome. AS plays vital roles in normal cellular function and development, and is widespread in human genome (Wang et al., 2008). Disruption or dysregulation of AS can lead to various diseases, including cancer, neurodegenerative diseases, cardiovascular diseases, and age-related diseases (Dlamini et al., 2015; Latorre and Harries, 2017; Du et al., 2021; Lv et al., 2021; Nikom and Zheng, 2023).

The serine/arginine-rich (SR) protein family, which contains one or two RNA recognition motifs (RRM) at the N-terminus and arginine/serine (RS)-rich domain at the C-terminus, is involved in the regulation of mRNA stabilization, translation, and splicing (Long and Caceres, 2009; Shepard and Hertel, 2009). The SR proteins and other splicing factors have been studied in several organisms using genetic editing approaches (Wang et al., 2016; Yuan et al., 2018). For example, disruption of the SR protein CeSF2/ASF in *Caenorhabditis elegans* results in growth defect and embryonic lethality (Longman et al., 2000). Mutation of SR-like RNA-binding protein slr1 in *Candida albicans* reduces its growth and filamentation, impairs its ability to damage host cells, and reduces the fungal virulence (Ariyachet et al., 2013). Ablation of SR-related protein RRM1 in *Trypanosoma brucei* results in cell cycle arrest and cell death (Levy et al., 2015).

Four SR splicing proteins (SR1, SR2, SR3, and SR4) are identified in *T. gondii* (Yeoh et al., 2015). Overexpression of SR3 causes growth defects and affects AS of over 1,000 genes in *T. gondii* (Yeoh et al., 2015). Given the important roles of SR proteins in *T. gondii* and other organisms, it is important to expand our understanding of the biological functions of other SR proteins in *T. gondii*. In this study, we investigated the biological roles of SR2 in *T. gondii* pathogenicity because its homologous protein in *Plasmodium falciparum* is essential for the parasite proliferation, and its overexpression affects the AS of *P. falciparum* genes (Eshar et al., 2012). CRISPR-Cas9 was used to establish knockout *T. gondii* strains, RH (type I) and Pru (type II), deficient in SR2. The resultant strains were used to identify the major phenotypic differences between the wild-type, the ∆*sr2*, and the complemented ∆*sr2*C strain to gain insights into the roles of SR2 in the parasite growth and pathogenicity. Our data showed that SR2 had no marked effect on the *in vitro* parasite growth and tachyzoite to bradyzoite differentiation, however it was significantly implicated in the virulence of *T. gondii* in mice.

## 2 Materials and methods

### 2.1 Mice

Specific-pathogen-free (SPF) female Kunming mice, between 6–8 weeks of age, were purchased from the Experimental Animal Center of Lanzhou Veterinary Research Institute, Chinese Academy of Agricultural Sciences (Lanzhou, China). All mice were acclimatized for one week prior to the study and had free access to water and food. All efforts were taken to minimize any suffering of the mice. Mice were immediately euthanized once they reach the humane endpoint. All experimental protocols were reviewed and approved by the Animal Research Ethics Committee of Lanzhou Veterinary Research Institute, Chinese Academy of Agricultural Sciences (Permit No. 2022–009).

### 2.2 Host cells and parasite strains

The tachyzoites used in this experiment, including those of the wild-type strains (RH∆*ku80* and Pru∆*ku80*, abbreviated as RH and Pru), knockout strains (RH∆*ku80*∆*sr2* and Pru∆*ku80*∆*sr2*, abbreviated as RH∆*sr2* and Pru∆*sr2*), complemented strains (RH∆*ku80*∆*sr2*C and Pru∆*ku80*∆*sr2*C, abbreviated as RH∆*sr2*C and Pru∆*sr2*C) and tagged strain (RH::SR2-6HA), were cultured by passage in human foreskin fibroblasts (HFFs, ATCC, Manassas, VA, United States). The cell cultures were maintained in Dulbecco’s Modified Eagle’s Medium (DMEM) supplemented with 2% fetal bovine serum (FBS), 10 mM HEPES (pH 7.2), 100 U/mL penicillin, and 100 μg/mL streptomycin at 37°C, humid atmosphere with 5% CO_2_ as previously described (Wang et al., 2020; Liang et al., 2021). Tachyzoites were isolated from heavily infected HFFs using a 27-gauge needle and filtered through a 5-μm polycarbonate membranes.

### 2.3 CRISPR-Cas9 mediated C-terminus epitope tagging

C-terminus epitope tagging was performed to examine the subcellular localization of SR2 as previously described (Cao et al., 2019; Liang et al., 2021). Briefly, specific CRISPR-Cas9 plasmid (30 μg) targeting SR2 C-terminus (after the stop codon), and a 42 bp homologous arm (20 μg) flanking the 6 × hemagglutinin (HA) tag and DHFR fragment amplified from pLIC-6HA-DHFR plasmid were co-transfected into purified tachyzoites. After treatment with 3 μM pyrimethamine, positive clones were identified by Western blotting (WB) and immunofluorescence assay (IFA). All plasmids, primers and guide RNA used in the study are summarized in Supplementary Table S1.

### 2.4 Construction of SR2 knockouts

The CRISPR-Cas9 mediated homologous recombination was used to produce SR2 knockouts as previously described (Wang et al., 2020). Briefly, the target guide RNA in the template plasmid pSAG1::CAS9-U6::sgUPRT was replaced by a specific guide RNA of SR2 to complete the construction of SR2-specific CRISPR plasmid. The 5′ and 3′ HR regions of SR2 amplified from *T. gondii* genomic DNA and DHFR fragment amplified from pUPRT-DHFR-D plasmid were ligated to the pUC19 fragment amplified from the pUC19 plasmid using the Clone Express II one-step cloning kit (Vazyme, Nanjing, China). The SR2-specific CRISPR plasmid (30 μg) and 5’HR-DHFR-3’HR homologous fragment (20 μg) amplified from total ligated product were transfected into the freshly purified tachyzoites. After selection with 3 μM pyrimethamine, single clones were isolated by limiting dilution and identified by PCR.

### 2.5 Generation of SR2-complemented strains

To complement SR2, coding sequence was integrated into the uracil phosphoribosyl transferase (UPRT) locus by homologous recombination as previously described (Wang et al., 2020). SR2 promoter amplified from *T. gondii* DNA and coding sequence of SR2 amplified from the cDNA of RH strain were fused with a fragment containing 3 × HA tag, 3’ UTR of DHFR, and a chloramphenicol acetyltransferase (Cat) cassette to produce complemented plasmids (pSR2::SR2::Cat). The corresponding fragments were amplified from pSR2::SR2::Cat plasmids, and co-transfected with the pSAG1::Cas9-U6::sgUPRT plasmid into the SR2 knockouts. Positive clones were selected by 20 μg/mL chloramphenicol acetyltransferase and identified using PCR and WB.

### 2.6 Immunofluorescence and Western blotting analysis

IFA was performed as previously described (Li D. et al., 2023; Li T. T. et al., 2023). Briefly, *T. gondii* infected samples were fixed with 4% paraformaldehyde (PFA) and permeabilized with 0.1% Triton-100 for 20 min at room temperature. After blocking with 3% bovine serum albumin (BSA) for 2 h at 37°C, the samples were incubated with primary antibodies, including rabbit anti-IMC1 (1:500), mouse anti-HA (1:500) (Thermo Fisher Scientific, Waltham, MA, United States) for 2 h at 37°C, followed by secondary antibodies, including Alexa Fluor 488 goat anti-rabbit IgG (H + L) (1:500), Alexa Fluor 594 goat anti-mouse IgG (H + L) (1,500) (Thermo Fisher Scientific, Waltham, MA, United States), and FITC-*Dolichos Biflorus* lectin (DBL, Vector Laboratories) at 37°C for 1 h. The samples were visualized using Leica Confocal microscope system (TCS SP8, Leica, Germany).

For the WB analysis, purified tachyzoites were lysed in RIPA buffer (Thermo Fisher Scientific, Waltham, MA, United States) on ice for 1 h to extract total protein. The lysates were separated by SDS-PAGE and transferred onto polyvinylidene fluoride (PVDF) membrane (Immobilon Millipore). Rabbit anti-aldolase (1:500), rabbit anti-HA (1:500), and goat anti-rabbit HRP (1,500) (Abcam, Cambridge, MA, United States) antibodies, 1% BSA working dilution, were used for WB analysis. ECL chemiluminescent reagent (Thermo Fisher Scientific, Waltham, MA, United States) was used to detect the signal and images were captured with Minichemi 610 chemiluminescent imager (Bio-Rad Laboratories, Hercules, CA, United States).

### 2.7 Plaque assay

To assess whether the absence of SR2 affects the lytic cycle of *T. gondii*, confluent HFFs grown on 12-well tissue culture plates (Thermo Fisher Scientific, Waltham, MA, United States) were infected by 500 freshly lysed tachyzoites per well for seven or nine days. After washing, five times with phosphate-buffered saline (PBS), HFFs were fixed with 4% PFA for 30 min and stained with 0.2% crystal violet for 20 min at room temperature. The size and number of plaques were examined with a scanner (Perfection V700 Photo) and analyzed by Image J.

### 2.8 Invasion assay

About 10^6^ freshly egressed tachyzoites were allowed to invade HFFs for 30 min and fixed with 4% PFA for 20 min. The infected cells were subsequently incubated with antibodies, including primary antibody, mouse anti-SAG1 antibody (1:500), and secondary antibody, Alexa Fluor 594 goat anti-mouse IgG (H + L) antibody (1:500). After three washes with PBS, samples were permeabilized with 0.1% Triton-100 and sequentially stained with rabbit anti-GAP45 antibody (1:500) and Alexa Fluor 488 goat anti-rabbit IgG (H + L) antibody (1,500). IFA was used to examine the parasite’s invasion efficiency by evaluating the ratio of intracellular tachyzoites (green-stained minus red-stained) and total tachyzoites (green-stained).

### 2.9 Intracellular replication assay

To evaluate the role of SR2 in the replication of *T. gondii*, 10^5^ freshly egressed tachyzoites were allowed to infect HFF monolayers on 12-well tissue culture plates for 1 h, and then washed with DMEM to remove extracellular tachyzoites. The plates were incubated at the same conditions for another 23 h, fixed with 4% PFA and then incubated with antibodies, including mouse anti-SAG1 (1:500), and Alexa Fluor 488 goat anti-mouse IgG (H + L) (1:500). The number of tachyzoites in at least 200 parasitophorous vacuoles (PVs) was counted.

### 2.10 Egress assay

To investigate whether SR2 is involved in the egress of *T. gondii*, 10^5^ freshly egressed tachyzoites were added to HFF monolayers for 1 h at 37°C and 5% CO_2_, and the un-attached tachyzoites were washed with DMEM. The samples were incubated for another 36 h and then treated with 3 μM calcium ionophore A23187 (Sigma, Burlington, MA, United States) for 2 min. The infected cells were fixed with 4% PFA and the proportion of egressed versus not egressed tachyzoites was examined microscopically to evaluate the egress efficiency as previously described (Liang et al., 2021).

### 2.11 Examining the role of SR2 in acute and chronic infection

Female mice (6 mice/group) were infected by intraperitoneal (i.p.) injection with 100 tachyzoites of RH, RH∆*sr2* and RH∆*sr2*C or 200 tachyzoites of Pru, Pru∆*sr2* and Pru∆*sr2*C to evaluate the impact of SR2 on acute and chronic *T. gondii* infection, respectively. A plaque assay was performed to ascertain the viability of the tachyzoites used in mouse infection. Mice were monitored twice a day for 30 days and euthanized once they reach the humane endpoint (loss of ≥20% of initial body weight). The brains of mice infected by Pru, Pru∆*sr2* and Pru∆*sr2*C were collected at 30 days after infection and homogenized with 1 mL PBS to count the number of brain cysts as previously described (Zheng et al., 2023). The experiments were repeated two independent times.

### 2.12 Determination of antibody levels induced by RH∆*sr2* strain

Enzyme-linked immunosorbent assay (ELISA) was used to evaluate concentration of antibodies in the serum of survived mice infected by RH∆*sr2* strain as previously described (Wang et al., 2017). In brief, a 96-well plate (Thermo Fisher Scientific, Waltham, MA, United States) was coated overnight with soluble tachyzoite antigen (STAg), blocked with 5% BSA, and then incubated with mouse serum as primary antibodies (1:50), and secondary antibodies, including horseradish-peroxidase-conjugated goat anti-mouse IgG (1:250), anti-mouse IgG1 (1:500), and IgG2a (1:500) (Abcam, Cambridge, MA, United States). After addition of 2% sulfuric acid, the absorbance was read at 450 nm using iMark microplate Absorbance reader (Bio-Rad, Hercules, CA, United States). To assess the protective efficacy against *T. gondii* re-infection, mice that survived infection by RH∆*sr2* were challenged, 45 days after infection, with 500 RH tachyzoites. Another group of mice was injected with 500 RH tachyzoites and served as a control. All mice were monitored twice daily.

### 2.13 *In vitro* bradyzoite differentiation

For bradyzoite differentiation, Pru, Pru∆*sr2* and Pru∆*sr2*C tachyzoites were induced under alkaline conditions as previously described (Wang et al., 2022). Briefly, tachyzoites were allowed to invade HFF monolayers for 4 h at 37°C with 5% CO_2_, and then cultured in RPMI-HEPES medium (pH 7.8, pH 8.0, and pH 8.2) for 48 h at 37°C without CO_2_. The medium was replaced every day to maintain the alkaline condition of the culture. IFA was performed to observe the proportion of parasites stained with rabbit anti-IMC1 (1:500) and Alexa Fluor 594 goat anti-rabbit IgG (H + L) (1:500) and the parasite cysts were stained with FITC-DBL.

### 2.14 Bioinformatic analysis of *Toxoplasma gondii* SR2

The data about *T. gondii* SR2, including amino acid sequence, signal peptide and transmembrane domain, were retrieved from ToxoDB.^1^ The domains of SR2 protein were analyzed by using ExPASy Server^2^ and relevant reference as previously described (Wan et al., 2019). The 3D structure of SR2 was constructed using the SWISS-MODEL server,^3^ based on the crystal structure of *T. gondii* RNA recognition motif-containing protein TGP89_217540 (PDB: A0A086K082.1.A). BLAST search for homologous proteins of SR2 was performed and phylogenetic tree based on amino acid sequence of SR2 protein was constructed by maximum likelihood method using MEGA-X software.

### 2.15 Statistical analysis

Statistical analysis was performed using GraphPad Prism software (version 8.01, GraphPad Software, Inc). One-way ANOVA and two-tailed, unpaired Student *t*-test analyses were used for comparisons between three or more groups or between two groups, respectively. The experiments were repeated three independent times. The results are shown as mean ± standard deviation (SD). The level of statistically significant differences is indicated on the figures by asterisks: *, *p* < 0.05; **, *p* < 0.01; ***, *p* < 0.001; ****, *p* < 0.0001.

## 3 Results

### 3.1 Characterization and localization of SR2

**Figure 1:**
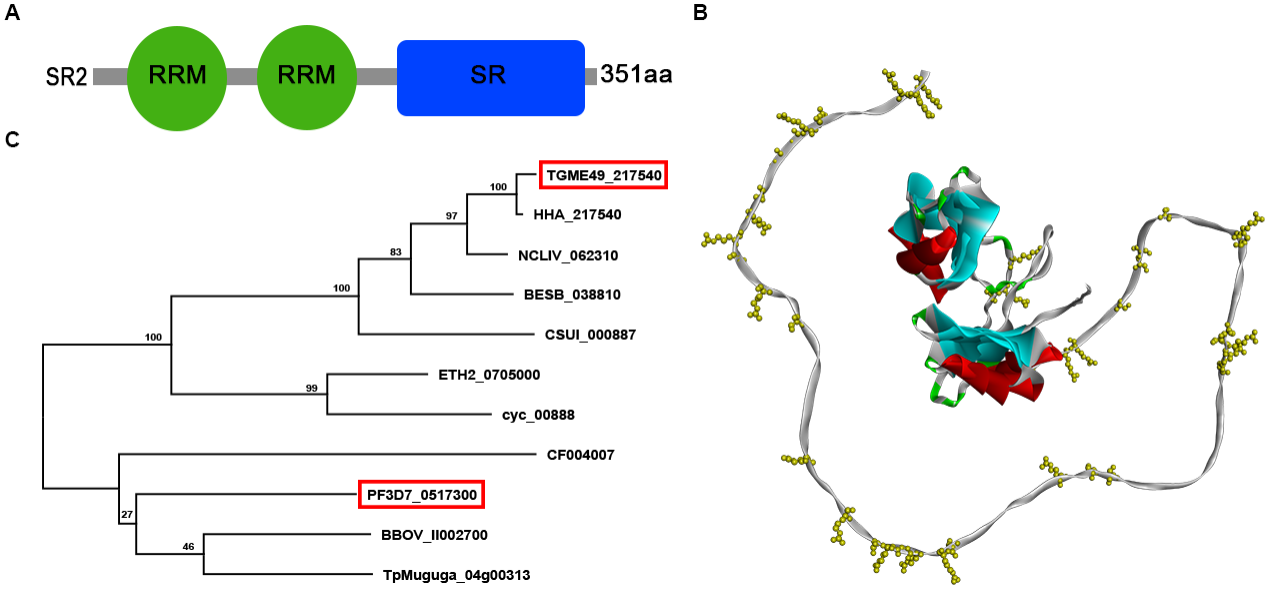
Characterization of the splicing factor SR2 in *Toxoplasma gondii*. **(A)** Domain analysis of splicing factor SR2 using ExPASy server. SR2 protein has two RRM domains (Green box) at the N-terminus and one RS domain (blue box) at the C-terminus. **(B)** Schematic representation of the 3D structure of the SR2 protein using SWISS-MODEL server based on the crystal structure of *T. gondii* RNA recognition motif-containing protein TGP89_217540 (PDB: A0A086K082.1.A). **(C)** Based on the amino acid sequence of SR2, phylogenetic tree of SR2 protein was constructed by maximum likelihood method using MEGA-X software.

**Figure 2:**
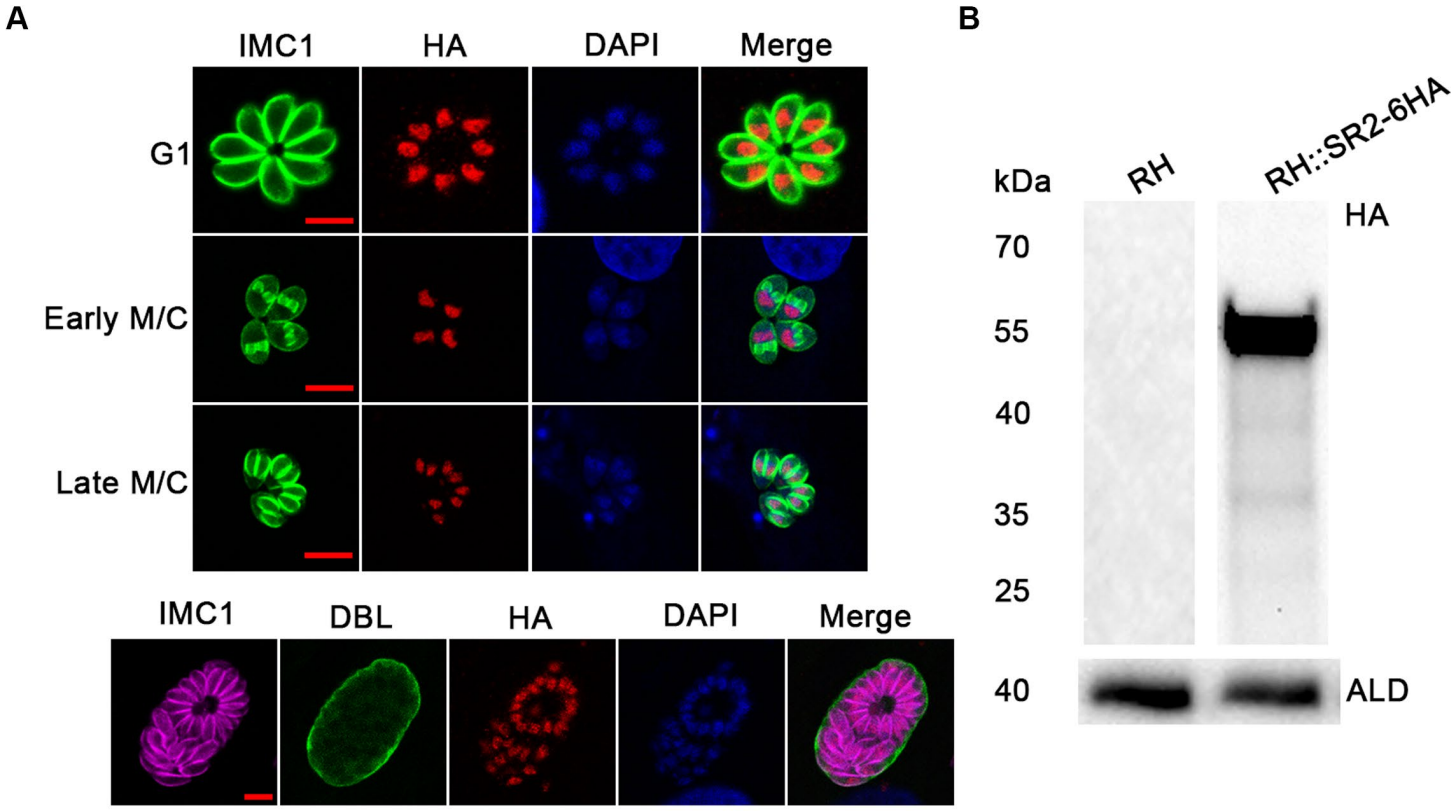
Subcellular localization and expression of SR2 in *T. gondii*. **(A)** Subcellular localization of SR2 in tachyzoites and bradyzoites. Rabbit anti-IMC1, mouse anti-HA and DAPI were used to stain the inner membrane complex (green), localize the tagged protein (red) and stain the nucleus (blue), respectively. Scale bars, 5 μm. IFA showed that SR2 in tachyzoites and bradyzoites was localized to the nucleus. **(B)** Western blotting confirmed the correct expression of SR2 coding region fused with 6HA tag as evidence by detection of a ~ 52 kDa band. Anti-aldolase (ALD) was regarded as a loading control.

According to the ExPASy Server,^4^ the predicted SR2 protein (TGME49_217540) consists of 351 amino acids and does not have a signal peptide or a transmembrane domain, but has two RRM domains at the N-terminus and one RS domain at the C-terminus (Figure 1A; Supplementary Figure S1). Based on the Swiss model, the structure of SR2 protein conformed to the classical structural characteristics of the SR protein family (Figure 1B) (Shepard and Hertel, 2009). Phylogenetic analysis showed that SR2 had high homology to the alternative splicing factor SR1 (PfSR1), which plays an essential role in the proliferation of *P. falciparum* (Figure 1C) (Eshar et al., 2012). To explore the subcellular localization of SR2, a 6 × HA epitope tag was successfully inserted into the C-terminus of SR2. IFA showed that SR2 was localized in the nucleus in the tachyzoite and bradyzoite stages (Figure 2A). WB analysis showed that cell lysates of 6 × HA tagged strain expressed the fusion protein and its size was ~52 kDa, which conforms with the predicted size of the protein (Figure 2B).

### 3.2 Successful disruption and complementation of SR2 in *Toxoplasma gondii* RH and Pru strains

**Figure 3:**
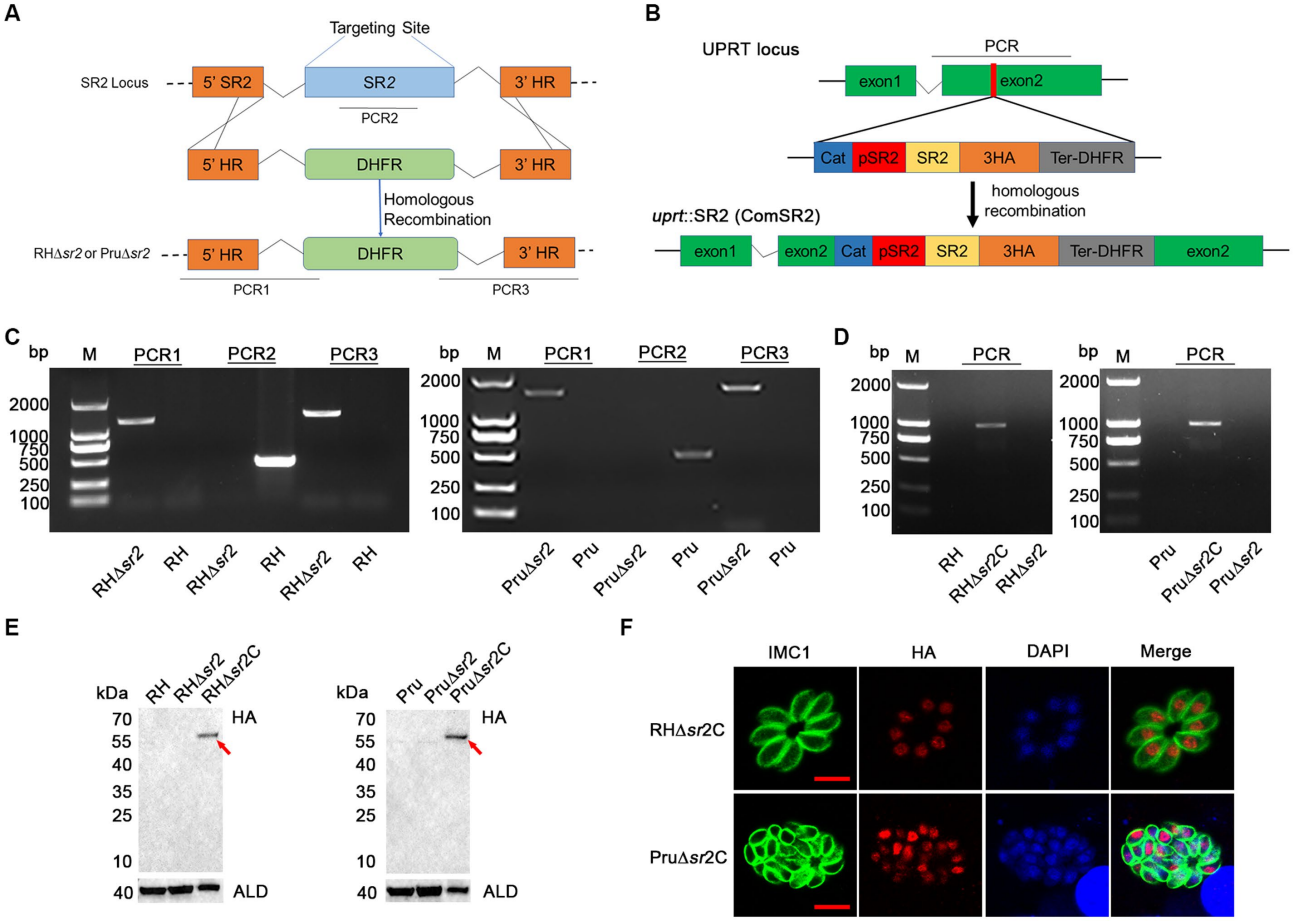
Generation of ∆*sr2* and ∆*sr2*C in type I RH strain and type II Pru strain. **(A)** Schematic diagram of CRISPR-Cas9 mediated homologous recombination. **(B)** Schematic representation of the insertion of coding region of SR2 fused with 3 × HA tag driven by SR2 promoter at the UPRT locus of ∆*sr2* strains. **(C)** Confirmation of the construction of ∆*sr2* strains by PCR. PCR2 was used to determine whether SR2 was deleted, and PCR1 and PCR3 were used to determine whether 5’ HR-DHFR-3’ HR fragment was successfully inserted into the target gene sequence. **(D-F)** Identification of ∆*sr2*C strains by PCR, IFA, and Western blotting analyses. PCR and Western blotting analyses showed that the expected fragments were amplified from ∆*sr2*C strains of type I RH and type II Pru, but not from wild-type and ∆*sr2* strains. IFA analysis showed the successful expression of the coding region of SR2 fused with 3HA tag at the UPRT locus of ∆*sr2* strains, confirming that SR2 was localized to the nucleus. Scale bars, 5 μm.

To investigate the biological function of SR2, 5′ HR-DHFR-3′ HR homologous fragment was used to disrupt coding region of SR2 (Figure 3A). The successful deletion of SR2 gene was confirmed using PCR2, where no bands were amplified in the ∆*sr2* strains, however, ~500 bp bands were detected in the wild-type strains. The correct replacement of the 5′ HR-DHFR-3′ HR fragment was confirmed by the amplification of ~1,000–1,500 bp bands from the ∆*sr2* strains by PCR1 and PCR3. These results showed that SR2 was successfully knocked out in type I RH strain and type II Pru strain (Figure 3C). ∆*sr2* complemented strains were constructed to further elucidate the function of SR2 (Figure 3B). PCR and WB analyses revealed that bands were amplified in the ∆*sr2*C strains, however these fragments were absent in the wild-type and ∆*sr2* strains (Figures 3D,E). Successful complementation of the coding region of SR2 fused with 3 × HA tag to ∆*sr2* strains was verified by IFA (Figure 3F).

### 3.3 Deletion of SR2 is dispensable for the growth or infectivity of *Toxoplasma gondii*

**Figure 4:**
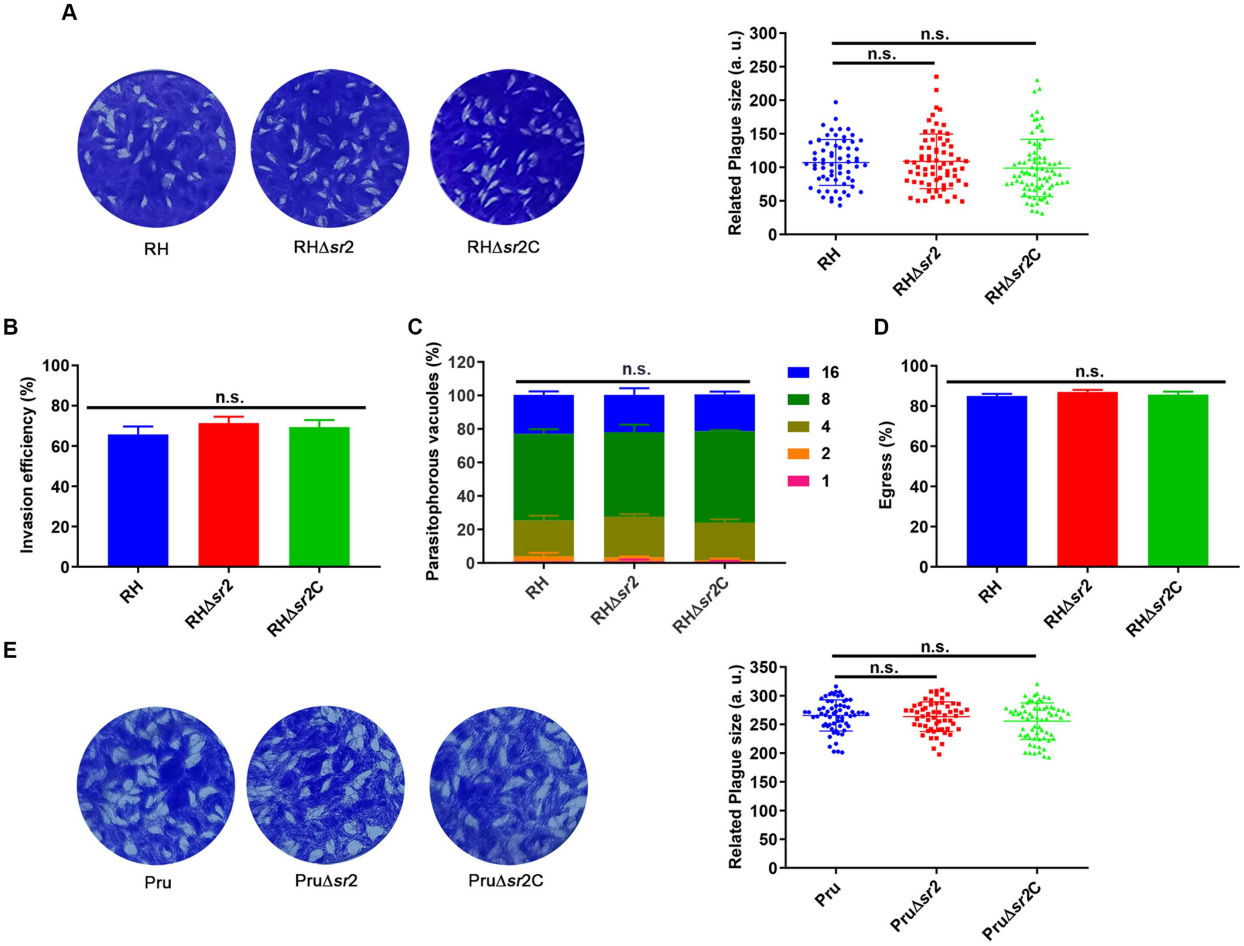
SR2 is dispensable for the growth of *T. gondii* in type I RH strain and type II Pru strain. **(A)** Representative plaques produced by RH, RH∆*sr2* and RH∆*sr2*C strains. No significant differences were detected in the number and size of plaques formed by RH, RH∆*sr2* and RH∆*sr2*C strains infecting HFFs for seven days. **(B-D)** SR2 was not essential for the invasion, intracellular replication, and egress of RH, RH∆*sr2* and RH∆*sr2*C tachyzoites. **(E)** Representative plaques formed by Pru, Pru∆*sr2* and Pru∆*sr2*C strains showing no significant differences in the number and size of plaques formed by Pru, Pru∆*sr2* and Pru∆*sr2*C strains nine days post-infection. n.s., not significant.

To assess the growth kinetics of *∆sr2* strains, plaque assay was performed. Seven or nine days after infection of HFFs, there were no significant differences in the size and number of plaques between the wild-type, the ∆*sr2* and the complemented strains (Figures 4A,E), indicating that absence of SR2 does not affect the lytic cycle of tachyzoites *in vitro*. Next, we performed the invasion, replication, and egress assay. For invasion assay, HFF monolayers were infected by RH, RH∆*sr2* and RH∆*sr2*C strains and the number of extracellular and intracellular tachyzoites was determined using fluorescence microscopy. The results showed that disruption of SR2 did not significantly affect the parasite invasion ability (Figure 4B). Intracellular replication assay was performed to evaluate proliferation efficiency of RH∆*sr2* strain based on the number of tachyzoites in each PV, 24 h post infection. RH∆*sr2* tachyzoites exhibited similar proliferation kinetics compared to the RH and RH∆*sr2*C tachyzoites (Figure 4C). To investigate the egress ability of RH∆*sr2* strain, HFF monolayers were infected for 36 h and treated with 3 μM calcium ionophore A23187. The results showed that RH∆*sr2* had the same egress efficiency as the RH and RH∆*sr2*C (Figure 4D). These results indicate that absence of SR2 does not cause significant alteration in the parasite’s ability for invasion, replication, or exit of the host cells, suggesting that SR2 does not play an important role in the parasite growth *in vitro*.

### 3.4 SR2 is important in acute and chronic toxoplasmosis of mice

**Figure 5:**
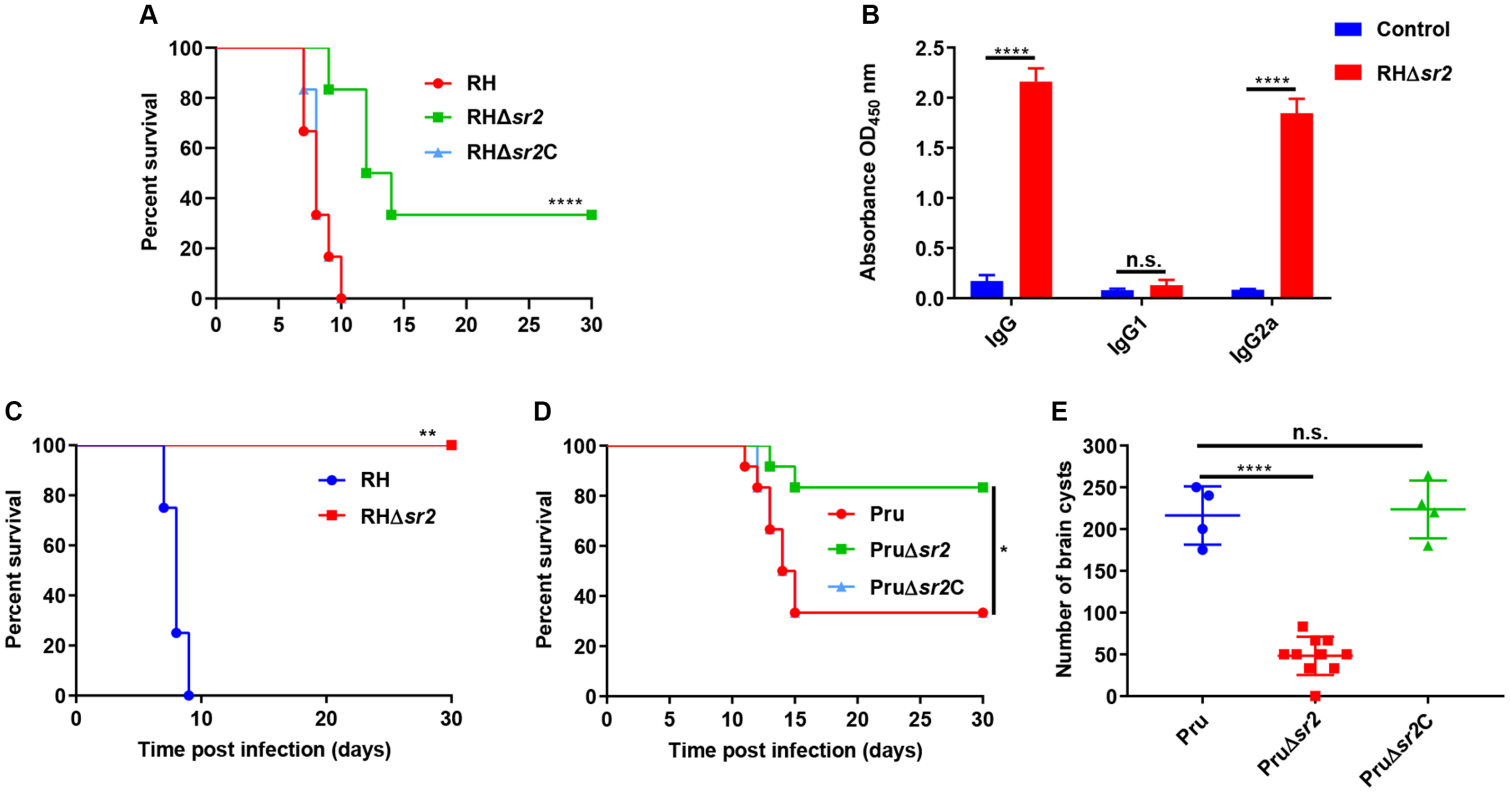
SR2 is important for *T. gondii* virulence. **(A)** Probability of survival of mice (6 mice/group) injected intraperitoneally (i.p.) with 100 tachyzoites of RH, RH∆*sr2* and RH∆*sr2*C. **(B)** Detection of high IgG and IgG2a antibody levels showed that the survived mice infected by viable RH∆*sr2* tachyzoites induced Th1 immune response. **(C)** Probability of survival of RH∆*sr2*-infected mice i.p. challenged, 45 days after infection, with 500 RH tachyzoites. **(D)** Survival of mice (6 mice/group) i.p. infected by 200 tachyzoites of Pru, Pru∆*sr2* and Pru∆*sr2*C. **(E)** The burden of brain cysts detected in mice infected by 200 tachyzoites of Pru, Pru∆*sr2* and Pru∆*sr2*C was determined at 30 days after infection. n.s., not significant. *, *p* < 0.05; **, *p* < 0.01; ***, *p* < 0.001; ****, *p* < 0.0001, compared with control.

To investigate the *in vivo* effect of the absence of SR2 on the parasite’s virulence, female mice (6 mice/group) were intraperitoneally infected by 100 tachyzoites of RH, RH∆*sr2* and RH∆*sr2*C or 200 tachyzoites of Pru, Pru∆*sr2* and Pru∆*sr2*C and the number of mice that exhibited humane endpoint was recorded. The results showed that disruption of SR2 in type I RH strain and type II Pru strains resulted in varying degrees of attenuated virulence (33.33% RH∆*sr2*; 0% RH or RH∆*sr2*C; 83.33% Pru∆*sr2*; 33.33% Pru or Pru∆*sr2*C). The complementation of SR2 gene rescued the virulence defect caused by SR2 deletion (Figures 5A,D).

To confirm that the survived mice are infected with viable RH∆*sr2* parasites, antibody levels produced by survived mice were determined using ELISA. The levels of IgG and IgG2a in RH∆*sr2*-infected mice were significantly higher than those in the control group (uninfected group) (*p* < 0.0001), but there were not significant changes in the IgG1 level (*p* > 0.05) (Figure 5B). The IgG2a/IgG1 ratio suggests a Th1 immune response. To investigate immune protection conferred by RH∆*sr2* strain, survived mice were challenged with 500 RH tachyzoites 45 days after initial infection. The results showed that mice infected by RH∆*sr2* tachyzoites survived the challenge with wild-type RH tachyzoites for 30 days, while mice in the uninfected group reached the human endpoint within nine days (*p* < 0.01), suggesting that immune response produced by the survived mice was effective in protecting against virulent infection (Figure 5C).

To investigate the role of SR2 in chronic infection, the number of brain cysts was quantified in mice infected by 200 tachyzoites of Pru, Pru∆*sr2* and Pru∆*sr2*C 30 days after infection. The results revealed significant reduction of ~80% in the brain cyst burden in mice infected by Pru∆*sr2* strain (46 ± 25 cysts/brain) compared to those infected by Pru (216 ± 35 cysts/brain) or Pru∆*sr2*C strains (224 ± 35 cysts/brain) (*p* < 0.0001) (Figure 5E), indicating that deletion of SR2 causes severe disruption of cyst formation.

### 3.5 Deletion of SR2 does not affect the bradyzoite differentiation *in vitro*

**Figure 6:**
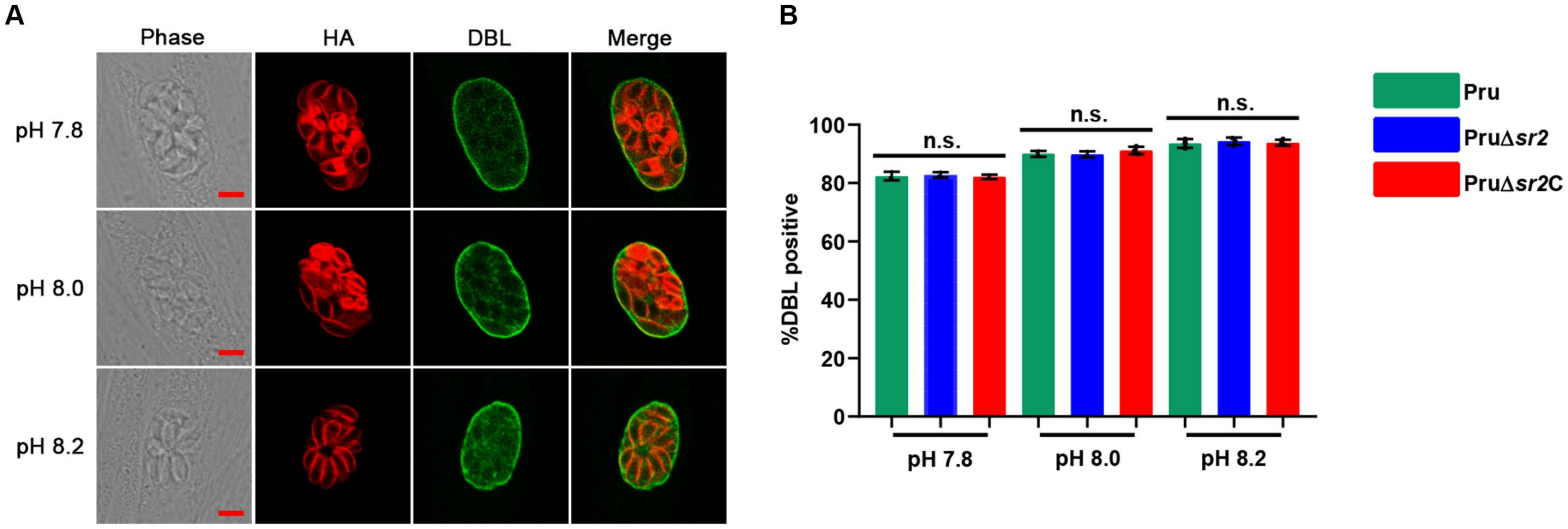
Deletion of SR2 does not affect tachyzoite to bradyzoite differentiation *in vitro*. **(A)** Pru, Pru∆*sr2* and Pru∆*sr2*C tachyzoites were allowed to invade HFFs for 4 h under normal culture conditions and then incubated in the absence of CO_2_ and under different alkaline stress conditions (pH 7.8, 8.0 and 8.2) for additional 48 h. Rabbit anti-IMC1 and FITC-DBL were used to stain the parasites (red) and cyst wall of bradyzoite (green), respectively. Scale bars, 5 μm. **(B)** Tachyzoite to bradyzoite conversion efficiency of Pru, Pru∆*sr2* and Pru∆*sr2*C under different alkaline conditions. No significant differences were detected in the proportion of DBL positive vacuoles of Pru, Pru∆*sr2* and Pru∆*sr2*C.

In view of the severe defects in cyst formation caused by disruption of SR2 *in vivo*, Pru, Pru∆*sr2* and Pru∆*sr2*C strains were exposed to different alkaline conditions (pH 7.8, pH 8.0, and pH 8.2) to study whether SR2 affects tachyzoite to bradyzoite transformation *in vitro*. The tachyzoite to bradyzoite transformation efficiency was evaluated by counting cyst wall stained with FITC-DBL using fluorescence microscopy. Surprisingly, the results showed that tachyzoite to bradyzoite conversion efficiency of Pru∆*sr2 in vitro* was not significantly different under different alkaline conditions compared with Pru or Pru∆*sr2*C strains, indicating that SR2 does not contribute to the tachyzoite to bradyzoite conversion *in vitro* (Figures 6A,B).

## 4 Discussion

The success of *T. gondii* lies in its remarkable ability to respond and adapt to various ecological and physiological niches, which involves orchestrating the transcription of many genes. As key regulators of AS, SR proteins are involved in key processes such as splicing, mRNA export, mRNA stability, and translation (Huang and Steitz, 2005). The multi-faceted roles of SR proteins and the fact that disruption of *P. falciparum* SR1 and *T. gondii* SR3 results in significant growth defect of these parasites (Eshar et al., 2012; Yeoh et al., 2015) inspired us to investigate the function of SR2 protein in *T. gondii*.

SR2 belongs to a family of multi-domain RNA binding proteins that regulate alternative splicing of genes involved in many biological processes (Huang and Steitz, 2005). SR proteins harbor structural modules for RNA recognition at the amino terminus, such as RRM domains and a RS domain at the carboxyl terminus required for protein–protein interaction (Shepard and Hertel, 2009). SR proteins are located in the nucleus and mainly localize to a subnuclear region in a process mediated by a nuclear localization signal, mainly the RS domain (Cáceres et al., 1997). To investigate the subcellular localization of SR2 in *T. gondii*, a 6 × HA-tag was inserted at the C-terminus of SR2 in RH strain and IFA analysis showed that SR2 was localized to the parasite nucleus in tachyzoites and bradyzoites, suggesting that SR2 is a SR protein.

Next, we investigated the role of the splicing factor SR2 in the growth and pathogenicity of *T. gondii*. *T. gondii* proliferation is based on successive lytic cycles involving the parasite invasion, intracellular proliferation, and egress of the host cell (Hortua Triana et al., 2018; Sanchez and Besteiro, 2021). Thus, we examined the effect of deletion of SR2 in type I RH strain and type II Pru strain on the parasite ability to invade, replicate and exit the host cells. Consistent with a previous report on the CRISPR-Cas9 phenotypic value of SR2 (Sidik et al., 2016), disruption of SR2 in type I RH strain and type II Pru strain did not significantly affect the parasite growth (based on the size and number of plaques produced by the parasite), invasion ability, intracellular replication efficiency, and egress ability.

Splicing factors have been shown to mediate the virulence of various pathogens. For example, KH-QUA2 (84KQ) motifs at the C-terminus of *Entamoeba histolytica* U2AF2 play roles in splicing of several virulence genes, and absence of 84KQ motifs alters splicing and reduces the parasite virulence (González-Blanco et al., 2022). The deletion of *Ustilago maydis Rrm75*, which encodes three RRM domains and glycine-rich repeats, fails to produce splicing variants at the 3′-alternative splicing locus of the third exon through AS, resulting in abnormal growth and virulence phenotypes (Rodríguez-Kessler et al., 2012). Therefore, we hypothesized that deletion of SR2 may influence the pathogenicity of *T. gondii*. We explored the role of splicing factor SR2 in the virulence of *T. gondii* during acute and chronic infection in mice. The results showed that the virulence of type I RH strain and type II Pru strain lacking splicing factor SR2 resulted in varying degrees of attenuation. The different attenuated virulence may be attributed to the distinct inherent differences in the virulence between type I RH strain and type II Pru strain (Howe and Sibley, 1995). These results show the adverse effect of SR2 deletion on the parasite virulence, which may be caused by dysregulated splicing. The mechanism by which SR2 affects the virulence of *T. gondii* remains to be investigated.

Notably, we observed a significant reduction of brain cyst burden in mice infected by Pru∆*sr2* compared with mice infected by Pru or Pru∆*sr2*C. However, the same strains did not exhibit significant differences in the tachyzoite to bradyzoite differentiation rate *in vitro*. This paradox could suggest the presence of host immune or molecular factors that contribute to reduction in tachyzoite to bradyzoite conversion in mice, but are lacking in the cell culture (Li D. et al., 2023; Li T. T. et al., 2023). However, the nature of this difference and the gene regulatory mechanisms controlling temporal expression of SR2 under *in vitro* and *in vivo* conditions remain to be elucidated.

The humoral immune response of mice infected by attenuated parasite strains can provide protection against re-infection by *T. gondii* (Zhang et al., 2018; Li D. et al., 2023; Li T. T. et al., 2023). Anti-*T. gondii* specific IgG2a and IgG1 isotypes levels are used as indicators for the balance between the Th1 and Th2 responses (Wang et al., 2017, 2018). Our results showed that mice which survived acute infection developed high level of IgG and IgG2a but low IgG1 level, consistent with Th1 immune response which is essential for protection against *T. gondii* re-infection (Rostamian et al., 2017; Wang et al., 2017). These results show that despite the low level of IgG1 antibodies, mice vaccinated by RHΔsr2 still have protective immunity against *T. gondii*, suggesting that IgG2a isotype correlated with increased protection against lethal *T. gondii* challenge than the IgG1 isotype, in agreement with previous studies (Wang et al., 2017, 2018).

## 5 Conclusion

We investigated the role of the splicing factor SR2 in the pathogenicity of *T. gondii*. Although disruption of SR2 did not significantly affect *T. gondii* growth and tachyzoite to bradyzoite differentiation *in vitro,* it markedly impaired the parasite virulence and cyst formation *in vivo*. These results suggest that SR2 deletion may affects the parasite virulence by disrupting the splicing regulatory network in *T. gondii*. Further investigations are required to elucidate the gene regulatory network controlling the expression and biological functions of SR2. Given the current situation of limited therapeutic options against *T. gondii* infection, searching for more effective drugs is needed to improve clinical anti-*T. gondii* chemotherapeutics. In this regard, screening and testing functional inhibitors against *T. gondii* SR2 is worthwhile.

## Data availability statement

The original contributions presented in the study are included in the article/Supplementary material, further inquiries can be directed to the corresponding authors.

## Ethics statement

The animal study was approved by the Animal Research Ethics Committee of Lanzhou Veterinary Research Institute, Chinese Academy of Agricultural Sciences. The study was conducted in accordance with the local legislation and institutional requirements.

## Author contributions

X-JW: Data curation, Formal analysis, Investigation, Methodology, Writing – original draft, Writing – review & editing. JG: Formal analysis, Investigation, Methodology, Validation, Writing – original draft. X-NZ: Formal analysis, Investigation, Methodology, Writing – review & editing. HE: Conceptualization, Resources, Visualization, Writing – review & editing. T-TL: Investigation, Methodology, Writing – review & editing. Y-JK: Investigation, Writing – original draft. MW: Conceptualization, Funding acquisition, Project administration, Resources, Supervision, Writing – review & editing. X-QZ: Conceptualization, Funding acquisition, Project administration, Resources, Supervision, Writing – review & editing.

## Footnotes

^1^ http://toxodb.org

^2^ https://www.expasy.org

^3^ https://swissmodel.expasy.org

^4^ https://www.expasy.org
