# Supplementary material for: The splicing factor SR2 is an important virulence factor of Toxoplasma gondii
Source: Front Microbiol. 2023 Nov 23;14:1302512. doi: 10.3389/fmicb.2023.1302512 (PMC10701758; doi:10.3389/fmicb.2023.1302512)
Supplement: Supplementary file 1 [file Data_Sheet_1.PDF]

MFGGDRSAAGGGGSGRIYIGSLPGEYTDKDVEREFEKFGKIIKLEFKRTVSGAGYCFLEYA
DPRDARDAIAQLHGRPPPGMRGAAPLRVEIPLARSSRPDGFGEEALPRGLAGRRGRFVLE
VRGLPPSGSWQDLKDHFRGIGDVGFAEVRKDPDAPDSVMGKVSFFSKRDMMEAIEVLDG
STFRSHEGEKSRISVREKRAPGGRGGRSDDAHDAAAYDTDARGGRRTKYGSLYESSNGG
YSTGGADAYRNSIEMMDPSSRGRSGGPGAGGYYGLHPTGSPSPPRGHAAYTENNRRGRS
RSGERGPPTRGAYGAYGPPASYYDRIGTSPRCPRLSMERGSRRFDDWTDRRRC

FIGURE S1. Amino acid sequence analysis of SR2. Results showed SR2 has two RNA recognition motif (RRM) domains at the N-terminal (amino acids 15-94 and 118-198) and one SR domain at the C-terminal. The RRM domains are highlighted in yellow, while each arginine (R) and serine (S) within the SR domain is annotated in green.
