# Supplementary material for: The splicing factor SR2 is an important virulence factor of Toxoplasma gondii
Source: Front Microbiol. 2023 Nov 23;14:1302512. doi: 10.3389/fmicb.2023.1302512 (PMC10701758; doi:10.3389/fmicb.2023.1302512)
Supplement: Supplementary file 2 [file Data_Sheet_2.PDF]

Table S1. All primers and plasmids used in this study

Primer	Sequence (5'-3')	Application
SgRNA-SR2-KO-F	GCTTATGACACAGATGCTCGGTTTTAGAGCTAGAAATAGC	Construction of SR2-specific CRISPR plasmid to construct ΔSR2 strains
SgRNA-R	AACTTGACATCCCCATTTAC	
U5-SR2-Gibson-F	GGTTTTCCCAGTCACGACGTTGGGGCTGTATCGACCAGGAAATC	Amplification of 5 'homologous arms of SR2 to construct SR2-pUPRT DHFR-D plasmid
U5-SR2-Gibson-R	GGATTTACAGCCTGGCGAAGCTTGTCTCCGCCGAACATTGTCTCAC	
U3-SR2-Gibson-F	CTATGCACTTGCAGGATGAATTCCTGGACAGACAGAAGGCGATGCT	Amplification of 5 'homologous arms of SR2 to construct SR2-pUPRT DHFR-D plasmid
U3-SR2-Gibson-R	GAGCGGATAACAATTTCACACCAAAGTTTGCCCTGCTTGGAGTA	
pUC19-Gbison-F	TGTGAAATTGTTATCCGCTC	Amplification of pUC19 fragment to construct SR2-pUPRT DHFR-D plasmid
pUC19-Gbison-R	AACGTCGTGACTGGGAAAACC	
DHFR-Gbison-F	AAGCTTCGCCAGGCTGTAAATCC	Amplification of DHFR fragment to construct SR2-pUPRT DHFR-D plasmid
DHFR-Gbison-R	GAATTCATCCTGCAAGTGCATAG	
U5-SR2-KZ-F	GGGGCTGTATCGACCAGGAAATC	Amplification of 5HR-DHFR-3HR homologous template of SR2 to construct ΔSR2 strains
U5-SR2-KZ-R	CCAAAGTTTGCCCTGCTTGGAGTA	
PCR1-SR2-F	ACAGATCAACCACGTGGACGTGAC	Detect the insertion of 5' homologous fragment of SR2 in PCR1
PCR1-DHFR-R	GCCAAAGTAGAAAGGAATTAGCAT	
PCR2-SR2-F	GGCATTGGCGATGTCGGTTTC	Detect the deletion of SR2 in PCR2
PCR2-SR2-R	CCCCTTCGGTTGTTTTCGGTGT	
PCR3-DHFR-F	TGACGCAGATGTGCGTGTATCCAC	Detect the insertion of 3' homologous fragment of SR2 in PCR3
PCR3-SR2-R	AAATGTCAGAGACGGAGACGCTCT	
pSR2-F	AGTGGAGGACGGGAATTCGGGCCCCAGAACAGACAGACAAGAACGCG	Amplification of SR2 promoter to construct PSR2::SR2::Cat plasmid
pSR2-R	CTTCTGTCTCCGCCGAACAT	
SR2-CDs-F	ATGTTCGGCGGAGACAGAAG	Amplification of SR2 CDs to construct PSR2::SR2::Cat plasmid
SR2-CDs-R	GGTCGAGCCCGAGCCCTTGCTAGCGCATCGCCTTCTGTCTGTCC	
3HA-Cat-Gbison-F	GCTAGCAAGGGCTCGGGCTCGACC	Amplification of 3HA-Cat fragment to construct PSR2::SR2::Cat plasmid
3HA-Cat-Gbison-R	GGGCCCGAATTCCCGTCCTCCACT	
UPRT-Pro-KZ-F	TCCTTTTATTCCAAGATCTGTGGCGTCTCGATTGTGAGGAAGTGGAGGACG GGAATTCG	Amplification of PSR2::SR2::Cat fragment to construct ΔSR2C strains
UPRT-Ter-KZ-R	AAACTGCCCGCAAGCCACTTTCCATCGACTCGCCAGCTAATACGACTCACT ATAGGGCG	
SgRNA-SR2-epitope tag	CAGACAGAAGGCGATGCTGAGTTTTAGAGCTAGAAATAGC	Construction of C-terminal SR2-specific CRISPR-Cas9 plasmid to construct C-terminal SR2 strains
SR2-HRF	GGAAGCAGGCGATTCGACGACTGGACAGACAGAAGGCGATGCGCTAGCAAGGGCTCGGG	Amplification of 6HA-DHFR fragment to construct C-terminal SR2 strains
SR2-HRR	CTACACGTGACCGATTTCAACTCTCTAGGTCTCTCACCTTCAATACGACTCACTATAGG	
